# Qualitative evaluation of pharmacological strategy for connective tissue diseases with Guillain-Barré syndrome: a systematic review

**DOI:** 10.3389/fimmu.2026.1684941

**Published:** 2026-03-18

**Authors:** Gaodi Yuan, Xiaomei Yang, Xue Xue, Jixian Yang, Zixia Yu, Lang Zhang, Jun Feng, Xiongyan Luo, Xiaoli Zheng, Anji Xiong

**Affiliations:** 1 Department of Rheumatology and Immunology, Beijing Anzhen Nanchong Hospital, Capital Medical University and Nanchong Central Hospital, The Affiliated Nanchong Central Hospital of North Sichuan Medical College, Nanchong, Sichuan, China; 2 Department of Rheumatology and Immunology, The First People's Hospital of Neijiang, Neijiang, Sichuan, China; 3 Nanchong Central Hospital, Nanchong Clinical Research Center, Nanchong, Sichuan, China; 4 Department of Otolaryngology, Beijing Anzhen Nanchong Hospital, Capital Medical University and Nanchong Central Hospital, The Affiliated Nanchong Central Hospital of North Sichuan Medical College, Nanchong, Sichuan, China; 5 Department of Rheumatology and Immunology, The Affiliated Hospital of North Sichuan Medical College, Nanchong, Sichuan, China; 6 Department of Rheumatology and Immunology, West China Hospital, Sichuan University, Chengdu, Sichuan, China; 7 School of Basic Medicine, Southwest Medical University, Luzhou, Sichuan, China

**Keywords:** case reports, connective tissue diseases (CTDs), Guillain-Barré syndrome (GBS), pharmacological treatment, systematic review

## Abstract

**Objective:**

To systematically evaluate the pharmacological management of Guillain-Barré syndrome occurring in the context of connective tissue diseases (CTD-GBS) and to investigate the relative efficacy of different treatment regimens on neurological outcomes.

**Methods:**

Case reports and series regarding CTD-GBS were systematically retrieved from PubMed, Embase, and Web of Science databases. A Generalized Linear Mixed Model (GLMM) was utilized to assess independent associations between treatment regimens and neurological improvement, adjusting for key covariates including mechanical ventilation (as a baseline severity marker) and the year of publication.

**Results:**

A total of 105 CTD-GBS patients were identified, with systemic lupus erythematosus (SLE) being the most prevalent subtype (n=73). Multivariable GLMM analysis suggested that, compared to intravenous immunoglobulin (IVIG) monotherapy, intensive combination regimens specifically glucocorticoids combined immunosuppressants (GC + IS) demonstrated a potential association with higher odds of clinical improvement (adjusted Odds Ratio [aOR] = 30.90; 95% CI: 6.58–145.00; p < 0.001). Mechanical ventilation was identified as an independent negative predictor of recovery (aOR = 0.43; p = 0.037), while the year of publication did not significantly influence outcomes (p = 0.344). Descriptive analysis within the SLE-GBS subgroup corroborated these trends, with the GC + IS regimen achieving a clinical improvement rate of 88.9%.

**Conclusion:**

Preliminary evidence suggests that intensive immunosuppressive combination therapy, notably GC + IS, may offer advantages over traditional IVIG monotherapy in improving short-term neurological outcomes for CTD-GBS patients. However, given the reliance on retrospective case-based evidence and the potential for confounding by indication, these findings should be interpreted as hypothesis-generating clinical clues rather than definitive guidelines. Future large-scale, prospective studies utilizing standardized functional assessment scales are urgently required to validate these preliminary observations.

## 1 Introduction

Guillain-Barré Syndrome (GBS) is an infection-induced acute autoimmune neurological disorder characterized by demyelination or axonal damage to the peripheral nerves. Treatment strategies for pure GBS include intravenous immunoglobulin (IVIG) and plasma exchange (PE), both of which have been shown to be effective in patients with pure GBS (1). However, when GBS coexists with connective tissue diseases (CTDs), the pathogenesis and immune manifestations of the disease become more complex. Patients with CTDs and GBS (CTDs-GBS) exhibit acute and antibody-mediated neurological injury, persistent complement activation, and chronic inflammation, which limit the efficacy of conventional treatment strategies for GBS (2, 3).

Although specific treatment strategies for patients with CTDs-GBS have received significant attention in recent years, the studies have mainly focused on case reports or small-sample analyses and therefore lack a systematic assessment of the differences in efficacy and evidence-based medical rationale. This study aimed to systematically evaluate pharmacologic treatment strategies for CTDs-GBS, with a primary focus on the management of the neurological symptoms of GBS rather than systemic CTD activity. Therefore, we systematically evaluated differences in the efficacy of different treatment strategies for patients with CTDs-GBS through a systematic review and sought more effective treatment strategies.

## 2 Material and methods

This systematic review was conducted following the methodology recommended in the PRISMA guidelines.

### 2.1 Search strategy

**Figure 1:**
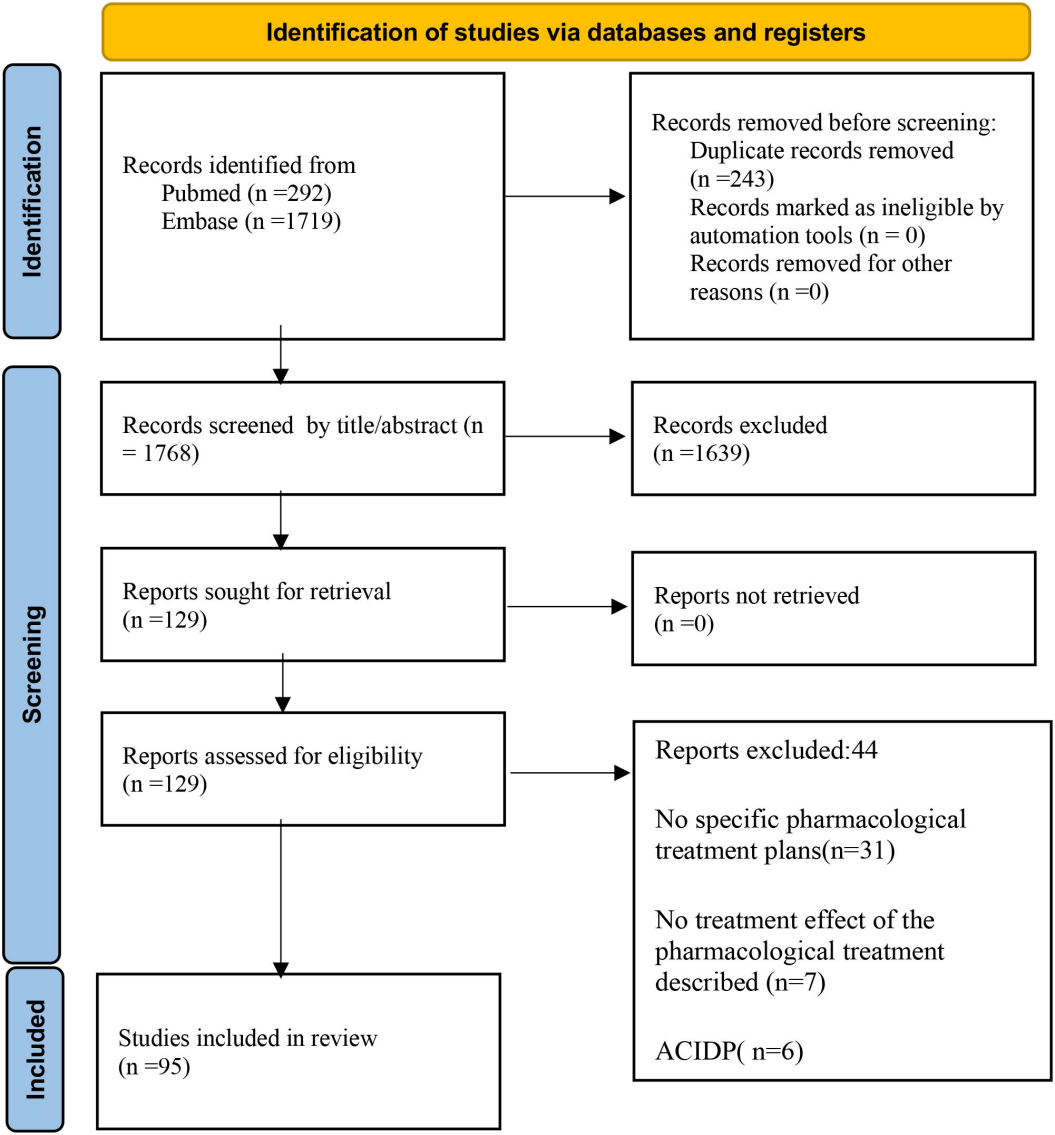
Flow diagram of the literature search and selection process.

A search of the PubMed, Embase, and Web of Science databases was carried out in English, with the most recent search date being November 13, 2024. And the date of publication of the identified studies ranged from 1980 to 2024. Keywords used for the search included “connective tissue diseases”, “Guillain-Barré syndrome”, “Guillain–Barre Syndrome”, “Guillain–Barre”, “acute autoimmune neuropathy”, “acute inflammatory demyelinating polyneuropathy”, “acute inflammatory demyelinating polyradiculoneuropathy”, “Miller–Fisher”, and related terms, with no language restrictions or other filtering conditions applied. Due to the rarity of cases, we aimed to capture all relevant studies to ensure the most comprehensive coverage of the available literature. All identified studies underwent strict quality assessment to ensure that only those meeting our predefined evaluation criteria were included in the analysis. Details of the search strategy can be found in the supplementary document. The Preferred Reporting Items for Systematic Reviews and Meta-Analyses (PRISMA) flowchart detailing the search strategy is shown in **Figure 1**.

### 2.2 Study selection

The inclusion criteria for studies were as follows: 1. Case reports, case series, and prospective or retrospective cohort studies. 2. Patients who met the diagnostic criteria for both CTD and GBS according to established diagnostic guidelines. Conversely, studies were excluded if they met any of the following criteria: 1. Studies that did not involve pharmacological treatment strategies. 2. Studies that did not address the effects of the pharmacological treatment.

### 2.3 Data extraction and quality assessment

The data extracted from each study included the first author’s name, year of publication, study region, age, sex, treatment strategy, clinical symptoms, laboratory test results, and changes in disease conditions. If multiple patients were reported across different studies or within the same group, the study participants were compared, and any duplicate data were excluded. Two authors (XX and YJX) independently extracted the data using standardized forms. They reviewed the information published in the original studies, and any discrepancies were resolved through consensus. The quality of each study was assessed using the “Tool for Evaluating the Methodological Quality of Case Reports and Case Series,” proposed by Murad et al. (4), based on previous criteria from the Pierson, Bradford Hill, and Newcastle-Ottawa scales. Each study was evaluated by the two authors (XX and YJX) across four domains: selection, ascertainment, causality, and reporting. This led to an overall assessment of each study (see **Supplementary Table 1**). The quality assessment included eight key exploratory questions with binary responses (yes/no) to identify any potential bias in the studies.

#### 2.3.1 Standardization of outcomes

To standardize heterogeneous outcome measures for systematic review, we uniformly defined treatment efficacy as the improvement or resolution of neurological symptoms (e.g., limb weakness, sensory disturbances, facial palsy, and respiratory dysfunction), relative to the baseline status immediately prior to each specific intervention. Since most case reports lacked standardized scoring systems, efficacy evaluation was based primarily on subjective clinical assessments. This approach ensures that the independent clinical response to each sequential treatment regimen is accurately captured.

Given the variability in disease presentation, outcome definitions, and follow-up duration across studies, this standardization approach was applied as a feasible strategy to facilitate the comparison of treatment responses across different CTD-GBS subtypes (e.g., systemic lupus erythematosus [SLE], Sjögren syndrome [SS], rheumatoid arthritis [RA], polymyositis [PM], or mixed connective tissue disease [MCTD]).

### 2.4 Statistical analysis

#### 2.4.1 Data independence and mixed-effects modeling

To account for the non-independence of observations arising from multiple treatment instances within individual patients (105 patients contributing 176 treatment instances), a Generalized Linear Mixed Model (GLMM) with a binomial distribution and logit link function was employed. Patient ID was included as a random effect to partition the variance attributable to within-subject correlations, thereby preventing sample size inflation and biased P-values. Statistical analyses were performed using R (version 2023.12.1 + 402).

#### 2.4.2 Covariate selection and data centering

The primary model evaluated the impact of different treatment regimens on clinical outcomes while adjusting for potential confounders. Fixed effects included: Treatment regimens (with IVIG monotherapy as the reference group). Baseline disease severity, defined by the requirement for mechanical ventilation and publication year, odds ratios (ORs) and 95% confidence intervals (CIs) were calculated, and a two-sided P < 0.05 was considered statistically significant.

#### 2.4.3 Efficacy attribution and sequential therapy logic

For patients receiving sequential therapies (e.g., initial failure of Strategy A followed by Strategy B), a specific efficacy attribution rule was applied to ensure logical rigor. Neurological improvement was attributed to the specific regimen initiated immediately prior to the observed recovery. If a previous treatment was discontinued due to lack of clinical response, it was recorded as no improvement, and the subsequent rescue therapy associated with recovery was recorded as improvement. This approach allows for a conservative estimation of each intervention’s independent contribution to the clinical outcome.

#### 2.4.4 Subgroup analysis and descriptive synthesis

Subgroup analyses were conducted based on CTD subtypes (e.g., SLE, SS, RA, PM, MCTD) and GBS variants (e.g., AIDP, AMAN, AMSAN, MFS). Given the inherent rarity of these subsets and the limited sample sizes (n < 3 in several categories), inferential statistical modeling was restricted to the total cohort to avoid overfitting and mathematical instability. For individual subgroups, descriptive statistics were used, with clinical outcomes presented as raw counts (n/N) and percentages to ensure data transparency and clinical relevance.

## 3 Results

### 3.1 Literature

A total of 95 studies met the inclusion criteria (5–99), with the majority corresponding to case reports. In this analysis, 105 patients were included. Since many patients received multiple treatments, each treatment was considered as a distinct unit of analysis, resulting in 176 treatment instances. The studies included in this analysis originated from several different countries, including China (8 cases), Japan (6 cases), United States (28 cases), United Kingdom (4 cases), and others. Due to the variation in geographic regions, ethnic and environmental factors may have influenced the disease progression and treatment outcomes, which may impact the generalizability of the findings. Further research is required to confirm the broader applicability of the findings. (A detailed distribution of the cases included can be found in the **Supplementary Material**.).

### 3.2 Study characteristics

A total of 105 patients with CTDs-GBS underwent 176 treatment instances, which were categorized into 13 different pharmacological modalities. The distribution of cases by primary disease was as follows: SLE: 73 patients, SS: 19 patients, RA: 7 patients, PM: 4 patients, and MCTD: 2 patients. The specific clinical features of patients with CTDs-GBS and Guillain-Barré syndrome related to SLE (SLE-GBS) are presented in **Table 1**.

**Table 1:** Main characteristics of patients with CTDs-GBS and SLE-related GBS.

Characteristics	Number(%)					
	CTDs-GBS	SLE-GBS	SLE-AIDP	SLE-AMAN	SLE-AMSAN	SLE-MFS
Total number of patients	105	73	18	10	17	4
Mean age, years (median; range)	33(38.6,11-82)	34(34.4,11-80)	32(36.2,17-75)	32(26.8,11-50)	31.5(41.4,19-77)	29(26.25,16-41)
Gender						
Female	78(74.3)	52(71.2)	12(63.2)	9(90.0)	12(70.6)	4(100.0)
Male	27(25.7)	21(28.8)	7(36.8)	1(10.0)	5(29.4)	0
Neurologic symptoms						
Four limbs weakness	66(62.8)	46(63.0)	11(57.9)	4(40.0)	13(76.5)	2(50.0)
Weakness limited to LLs	15(14.2)	13(17.8)	3(15.8)	4(40.0)	1(5.9)	2(50.0)
Sensory disturbances	31(29.5)	23(31.5)	7(36.8)	0	9(52.9)	0
Hyporeflexia or areflexia	51(48.6)	32(42.8)	9(47.4)	3(30.0)	11(64.7)	1(25.0)
Ataxia	10(9.5)	8(11.0)	1(5.3)	2(20.0)	1(5.9)	2(50.0)
facial paralysis	11(10.5)	8(11.0)	2(10.5)	2(20.0)	1(5.9)	1(25.0)
Ocular muscle paralysis	13(12.3)	12(16.4)	0	2(20.0)	4(23.5)	3(75.0)
diplopia	9(8.5)	7(9.6)	0	1(10.0)	2(11.8)	2(50.0)
Respiratory failure						
Non-invasive ventilation	8(7.6)	8(11.0)	1(5.3)	5(50.0)	1(5.9)	0
Invasive ventilation	34(32.3)	27(37.0)	7(36.8)	3(30.0)	9(52.9)	1(25.0)
Albumin-cytological dissociation						
N/A	22(20.9)	10(13.7)	2(10.5)	2(20.0)	1(5.9)	1(25.0)
Present	69(65.7)	52(71.2)	17(89.5)	6(60.0)	11(64.7)	1(25.0)
Absent	15(14.2)	11(15.1)	0	2(20.0)	5(71.4)	2(50.0)
Serologic tests						
Antinuclear antibody	63(60.0)	49(67.1)	13(68.4)	9(90.0)	10(58.8)	4(100.0)
Anti-double-stranded DNA antibody	51(48.6)	49(67.1)	15(78.9)	9(90.0)	8(47.1)	4(100.0)
Anti-Smith antibody	12(11.4)	9(12.3)	3(15.8)	2(20.0)	2(11.8)	2(50.0)
Anti-SSA antibody	25(23.8)	11(15.1)	2(10.5)	1(10.0)	2(11.8)	0
Anti-RNP antibody	11(10.5)	10(13.7)	2(10.5)	1(10.0)	2(11.8)	0
Anti-SSB antibody	10(9.5)	4(5.5)	1(5.3)	1(10.0)	0	0

CTDs, Connective tissue diseases; GBS, Guillain-Barré syndrome; SLE, systemic lupus erythematosus; AIDP, Acute Inflammatory Demyelinating Polyneuropathy; AMAN, Acute Motor Axonal Neuropathy; AMSAN, Acute Motor and Sensory Axonal Neuropathy; MFS, Miller Fisher Syndrome.

### 3.3 Multivariable analysis of treatment efficacy

#### 3.3.1 Primary findings from the GLMM

A Generalized Linear Mixed Model (GLMM) was utilized to evaluate the independent associations between treatment regimens and clinical outcomes. Compared to the reference group (IVIG monotherapy), several combination therapies were associated with significantly higher odds of neurological improvement. The GC + IS (Glucocorticoids + Immunosuppressants) regimen yielded an adjusted Odds Ratio (aOR) of 30.90 (95% CI: 6.58–145.00; p < 0.001). Other regimens demonstrating statistically significant associations included: PE + GC + IVIG: aOR = 26.30 (95% CI: 2.43–284.00; p = 0.007), PE + GC: aOR = 18.60 (95% CI: 2.99–116.00; p = 0.002), GC + IVIG + IS: aOR = 15.80 (95% CI: 3.24–76.70; p < 0.001), PE + GC + IS: aOR = 14.30 (95% CI: 3.31–61.90; p < 0.001).

#### 3.3.2 Impact of clinical covariates

The model identified mechanical ventilation as a significant negative predictor of clinical improvement (aOR = 0.43; 95% CI: 0.20–0.95; p = 0.037). In contrast, publication year (centered at 2000) was not significantly associated with the likelihood of recovery (aOR = 0.98 per year; p = 0.344).

#### 3.3.3 Comparison with monotherapies

After adjusting for baseline severity and publication year, monotherapies did not show statistically significant differences compared to IVIG monotherapy. Specifically, the aOR was 1.97 for GC alone (p = 0.298) and 2.22 for PE alone (p = 0.336).

#### 3.3.4 Sensitivity analysis

A sensitivity analysis was conducted by restricting the cohort to high-quality studies (n=84) after the exclusion of eleven cases identified as low quality based on the Murad et al. assessment tool. In this subgroup, the multivariable GLMM analysis demonstrated that the GC + IS regimen remained significantly associated with higher odds of clinical improvement compared to IVIG monotherapy (aOR = 30.20, 95% CI: 6.57–138.00, p < 0.001).Significant therapeutic effects were also observed for other combination regimens, including PE + GC + IS (aOR = 17.20, 95% CI: 3.65–81.50, p < 0.001), PE + GC (aOR = 13.90, 95% CI: 2.29–84.90, p = 0.004), and GC + IVIG + IS (aOR = 13.20, 95% CI: 2.76–62.80, p = 0.001).Regarding covariates, mechanical ventilation was identified as a significant independent predictor of poorer clinical outcomes (aOR = 0.43, 95% CI: 0.19–0.96, p = 0.040). The year of publication did not show a statistically significant association with clinical improvement in this high-quality subset (aOR = 0.97, 95% CI: 0.93–1.02, p = 0.223).

### 3.4 Pharmacologic treatment effects in patients with SLE-GBS

Within the SLE-GBS subgroup, 73 patients contributed 130 treatment instances. The clinical outcomes for the identified treatment regimens are summarized as follows (improvement/total instances). Monotherapies: improvement was observed in 3/24(12.5%) of IVIG, 3/8(37.5%) of PE, 2/17(11.8%) of GC, and 1/2(50.0%) of IS. Dual combinations therapies: improvement rates were 16/18(88.9%) of GC+IS, 2/5(40.0%) of PE+IVIG, 5/5(100.0%) of PE+IS, 4/6(66.7%) of PE+GC, and 4/15(26.7%) of GC+IVIG. Triple and other intensified therapies: improvement rates were 10/13(76.9%) of GC+IVIG+IS, 1/1(100%) of PE+GC+IVIG+IS, 2/3() of PE+GC+IVIG and 11/13(84.6%) of PE+GC+IS.

#### 3.4.1 Pharmacologic treatment effects in patients with Sjögren syndrome with GBS

Within the SS-GBS subgroup, 19 patients contributed 27 treatment instances. The clinical outcomes for the identified treatment regimens are summarized as follows (improvement/total instances). Monotherapies: improvement was observed in 4/9(44.4%) of IVIG, 0/3(0%) of PE, and 2/3(66.6%) of GC. Dual combinations therapies: improvement rates were 2/2(100%) of GC+IS, 1/1(100.0%) of PE+IS, 2/2(100%) of PE+GC, and 3/3(100%) of GC+IVIG. Triple and other intensified therapies: improvement rates were 1/1(100%) of GC+IVIG+IS, 1/2(50.0%) of PE+GC+IS, and 1/1(100%) of PE+GC+IVIG.

#### 3.4.2 Pharmacologic treatment effects in patients with rheumatoid arthritis with GBS

Within the RA-GBS subgroup, 7 patients contributed 9 treatment instances. The clinical outcomes for the identified treatment regimens are summarized as follows (improvement/total instances). Monotherapies: improvement was observed in 0/2(0%) of IVIG, and 3/3(100%) of GC. Dual combinations therapies: improvement rates were 0/1(0%) of GC+IS, 1/1(100%) of PE+GC, and 1/1(100%) of GC+IVIG. Triple and other intensified therapies: improvement rates were 1/1(100%) of PE+GC+IS.

#### 3.4.3 Pharmacologic treatment effects in patients with polymyositis with GBS

Within the PM-GBS subgroup, 4 patients contributed 5 treatment instances. The clinical outcomes for the identified treatment regimens are summarized as follows (improvement/total instances). Monotherapies: improvement was observed in 0/1(0%) of GC and 0/1(0%) of PE. Dual combinations therapies: improvement rates were 1/1(100%) of PE+GC, and 0/1(0%) of GC+IVIG. Triple and other intensified therapies: improvement rates were 1/1(100%) of PE+GC+IS.

#### 3.4.4 Pharmacologic treatment effects in patients with mixed connective tissue disease GBS

Within the MCTD-GBS subgroup, 2 patients contributed 2 treatment instances. The clinical outcomes for the identified treatment regimens are summarized as follows (improvement/total instances). Monotherapies: improvement was observed in 1/1(100%) of IVIG. Dual combinations therapies: improvement rate was 1/1(100%) of GC+IVIG.

### 3.5 Pharmacologic treatment effects in patients with CTDs with acute inflammatory demyelinating polyneuropathy

Within the CTDs-AIDP subgroup, 26 patients contributed 52 treatment instances. The clinical outcomes for the identified treatment regimens are summarized as follows (improvement/total instances). Monotherapies: improvement was observed in 1/9(11.1%) of IVIG, 0/5(0%) of PE, 2/4(50%) of GC, and 1/2(50.0%) of IS. Dual combinations therapies: improvement rates were 8/9(88.9%) of GC+IS, 2/2(100.0%) of PE+IS, 2/3(66.7%) of PE+GC, and 1/5(20%) of GC+IVIG. Triple and other intensified therapies: improvement rates were 1/2(50%) of GC+IVIG+IS, and 5/6(83.3%) of PE+GC+IS.

### 3.6 Pharmacologic treatment effects in patients with CTDs with acute motor axonal neuropathy

Within the CTDs-AMAN subgroup, 14 patients contributed 26 treatment instances. The clinical outcomes for the identified treatment regimens are summarized as follows (improvement/total instances). Monotherapies: improvement was observed in 3/6(50%) of IVIG, 1/3(33.3%) of PE, and 0/5(0%) of GC. Dual combinations therapies: improvement rates were 2/3(66.7%) of GC+IS, 2/2(100.0%) of PE+IS, 1/1(100%) of PE+IVIG and 1/3(33.3%) of GC+IVIG. Triple and other intensified therapies: improvement rates were 2/2(100%) of GC+IVIG+IS and 1/1(100%) of PE+GC+IVIG+IS.

### 3.7 Pharmacologic treatment effects in patients with CTDs with acute motor and sensory axonal neuropathy

Within the *CTDs-AMSAN* subgroup, 24 patients contributed 41 treatment instances. The clinical outcomes for the identified treatment regimens are summarized as follows (improvement/total instances). Monotherapies: improvement was observed in 1/10(10%) of IVIG, 1/1(100%) of PE, and 0/5(0%) of GC. Dual combinations therapies: improvement rates were 3/4(75%) of GC+IS, 1/3(33.3%) of PE+IVIG, 1/1(100.0%) of PE+IS, 2/2(100%) of PE+GC, and 2/4(50%) of GC+IVIG. Triple and other intensified therapies: improvement rates were 2/3(66.7%) of GC+IVIG+IS, 1/2() of PE+IVIG+GC and 5/6(83.3%) of PE+GC+IS.

### 3.8 Pharmacologic treatment effects in patients with CTDs with miller fisher syndrome

Within the CTDs-MFS subgroup, 6 patients contributed 12 treatment instances. The clinical outcomes for the identified treatment regimens are summarized as follows (improvement/total instances). Monotherapies: improvement was observed in 1/3(33.3%) of IVIG, 0/1(0%) of PE, and 1/2(50%) of GC. Dual combinations therapies: improvement rates were 1/1(100%) of GC+IS, and 1/2(50%) of GC+IVIG. Triple and other intensified therapies: improvement rates were 2/3(66.7%) of PE+GC+IS.

### 3.9 Pharmacologic treatment effects in patients with SLE-GBS

The results in Section 3.4 showed significant differences in the efficacy of the treatment strategies for patients with SLE-GBS. Thus, GBS can be further subdivided into several subtypes, such as AIDP, AMAN, AMSAN, and MFS, and the different subtypes differ in terms of pathomechanism, clinical manifestations, and prognosis. Therefore, it is important to explore the therapeutic response and outcomes of patients with SLE combined with different GBS subtypes to optimize treatment strategies. Consequently, we analyzed the differences in the effectiveness of treatment regimens for SLE combined with GBS across subtypes to provide a more precise basis for clinical decision-making.

#### 3.9.1 Pharmacologic treatment effects in patients with SLE with AIDP

Within the SLE-AIDP subgroup, 18 patients contributed 34 treatment instances. The clinical outcomes for the identified treatment regimens are summarized as follows (improvement/total instances). Monotherapies: improvement was observed in 1/6(16.7%) of IVIG, 0/3(0%) of PE, 0/2(0%) of GC, and 1/2(50.0%) of IS. Dual combinations therapies: improvement rates were 6/7(85.7%) of GC+IS, 2/2(100.0%) of PE+IS, 1/2(50%) of PE+GC, and 0/3(0%) of GC+IVIG. Triple and other intensified therapies: improvement rates were 1/2(50%) of GC+IVIG+IS, and 5/5(100%) of PE+GC+IS.

#### 3.9.2 Pharmacologic treatment effects in patients with SLE with AMAN

Within the SLE-AMAN subgroup, 10 patients contributed 19 treatment instances. The clinical outcomes for the identified treatment regimens are summarized as follows (improvement/total instances). Monotherapies: improvement was observed in 0/2(0%) of IVIG, 1/2(50%) of PE, and 0/4(0%) of GC. Dual combinations therapies: improvement rates were 2/2(100%) of GC+IS, 1/1(100%) of PE+IVIG, 2/2(100.0%) of PE+IS, and 1/3(33.3%) of GC+IVIG. Triple and other intensified therapies: improvement rates were 2/2(100%) of GC+IVIG+IS, and 1/1(100%) of PE+GC+IVIG+IS.

#### 3.9.3 Pharmacologic treatment effects in patients with SLE with AMSAN

Within the SLE-AMSAN subgroup, 17 patients contributed 33 treatment instances. The clinical outcomes for the identified treatment regimens are summarized as follows (improvement/total instances). Monotherapies: improvement was observed in 1/8(12.5%) of IVIG, 1/1(100%) of PE, and 0/5(0%) of GC. Dual combinations therapies: improvement rates were 2/3(66.7%) of GC+IS, 1/3(33.3%) of PE+IVIG, 1/1(100.0%) of PE+IS, 1/1(100%) of PE+GC, and 0/2(0%) of GC+IVIG. Triple and other intensified therapies: improvement rates were 2/3(66.7%) of GC+IVIG+IS, 1/2(50%) of PE+GC+IVIG and 4/4(100%) of PE+GC+IS.

#### 3.9.4 Pharmacologic treatment effects in patients with SLE with MFS

Within the SLE-MFS subgroup, 4 patients contributed 10 treatment instances. The clinical outcomes for the identified treatment regimens are summarized as follows (improvement/total instances). Monotherapies: improvement was observed in 0/2(0%) of IVIG, 0/1(0%) of PE, and 0/1(0%) of GC. Dual combinations therapies: improvement rates were 1/1(100%) of GC+IS, and 1/2(50%) of GC+IVIG. Triple and other intensified therapies: improvement rates were 2/3(66.7%) of PE+GC+IS.

## 4 Discussion

In this study, we systematically evaluated the efficacy of different treatment strategies for patients with CTDs-GBS to provide clinicians with recommendations on treatment options that can be drawn upon. It is important to emphasize that this study primarily evaluated the therapeutic efficacy on CTDs-GBS patients in terms of acute neurological symptoms. Efficacy assessment was primarily based on improvements in neurological symptoms such as limb weakness, sensory disturbances, facial paralysis, and respiratory dysfunction, rather than overall control of the underlying CTD.

Upon applying a GLMM to account for various clinical covariates (such as the requirement for mechanical ventilation and the year of publication), our preliminary findings suggest a potential trend: combination regimens specifically the GC and IS may offer advantages in improving clinical outcomes over traditional IVIG monotherapy. Although the GC + IS regimen yielded a statistically significant effect estimate (aOR = 30.90; 95% CI: 6.58–145.00; p < 0.001), these figures should be interpreted with caution as exploratory clinical references, given the low level of evidence and the substantial uncertainty reflected by the wide confidence intervals. To address potential biases inherent in case-based evidence, we further conducted a sensitivity analysis by restricting the cohort to high-quality studies. The stability of the results in this subset—most notably for the GC + IS regimen (aOR = 30.20; 95% CI: 6.57–138.00; p < 0.001)—demonstrates that the observed therapeutic advantage is robust and not driven by reports with lower methodological quality. Ultimately, combination therapy including immunosuppression may be associated with higher rates of short-term neurological improvement in published case reports; this hypothesis requires prospective confirmation. Nonetheless, this observation underscores that the immunopathological complexity of CTDs-GBS likely extends beyond the reach of conventional IVIG or PE alone, potentially justifying a more proactive immunosuppressive approach in clinical practice. While IVIG or PE are recommended for pure GBS, their limited success in CTD-GBS may stem from its immunological complexity (1–3, 100).

In addition to therapeutic strategies, our model highlighted a negative association between mechanical ventilation and clinical improvement (aOR = 0.43, p = 0.037). This observation further suggests that baseline disease severity may be a critical prognostic factor, an impact that appears to persist regardless of the specific intervention employed. Within our analysis, patients requiring respiratory support showed a likelihood of rapid improvement that was approximately halved. This trend underscores the potential importance of early, proactive intervention for high-risk individuals, aiming to forestall progression to mechanical ventilation a factor that appears to be a notable hurdle to favorable outcomes in our data. Interestingly, publication year showed no significant association with recovery (p = 0.344), which tends to support the notion that the observed benefits of combination regimens remain relatively stable across different medical eras rather than being solely a reflection of advances in supportive care.

In the descriptive analysis of the SLE-GBS subgroup, the distribution of clinical improvement appears to align with the trends identified in the overall GLMM model. Within this subgroup, treatment instances involving the GC + IS regimen showed a notably high proportion of clinical recovery (88.9%). While the descriptive nature of this sub-analysis precludes formal inferential testing, this observed trend provides preliminary, observational support for the potential advantages of intensive immunosuppression in the management of SLE-associated neurological complications.

For relatively rare CTD-GBS subtypes, such as those associated with Sjögren’s syndrome (SS), rheumatoid arthritis (RA), and polymyositis (PM), the extremely limited number of included cases restricts our findings to a preliminary synthesis of raw clinical responses. Although these small sample sizes are insufficient to support generalized therapeutic recommendations, initial observations suggest that the majority of cases achieving improvement similarly involved immunosuppressive interventions directed at the underlying primary disease. Consequently, these descriptive findings should be interpreted as clinical cues rather than definitive guidelines. They suggest that when managing such rare overlapping syndromes, clinicians may use the trends observed in this study as a reference while prioritizing individualized therapeutic weighing.

Overall, our review of published cases suggests that regimens including immunosuppression were more frequently associated with short-term neurological improvement than IVIG or glucocorticoids alone. However, given the low quality and heterogeneity of evidence, these findings should be considered hypothesis-generating rather than definitive clinical recommendations. Previous results suggest that CYC may be a beneficial option for treating CTDs-GBS (101), but studies comparing the efficacy of different immunosuppressants in patients with CTDs-GBS are scarce. Therefore, clinicians should select IS agents based on the condition of the individual patient and the side effects of the drug. For patients with mild disease, agents associated to lower toxicity such as HCQ or MMF may offer better clinical outcomes. For more severe cases requiring stronger immunosuppression, CYC, AZA, or MTX may be considered, but their potential side effects should be closely monitored (102–110).

Although our study offers valuable insights into treatment selection for patients with CTDs-GBS, several limitations must be acknowledged. First, a primary limitation of this study lies in the definition of treatment efficacy characterized as the improvement or disappearance of neurological symptoms. Since the source reports were largely case descriptions, this assessment relied heavily on subjective clinical judgment rather than standardized functional metrics (e.g., GBS disability scores) or predefined, fixed time points. Such a qualitative approach inherently introduces significant outcome heterogeneity and increases the risk of misclassification bias. Specifically, it remains challenging to distinguish between immediate, transient clinical improvements and sustained, long-term neurological recovery based solely on retrospective narratives. Consequently, the observed therapeutic effects should be interpreted as exploratory and hypothesis-generating, reflecting potential trends rather than definitive outcomes. To mitigate these inherent uncertainties, we employed a GLMM with Patient ID as a random effect to account for individual variability, yet we acknowledge that the lack of objective, longitudinal data remains a notable constraint. Second, the potential for confounding by indication must be acknowledged, as clinicians may have reserved more aggressive regimens (e.g., PE + IS) for patients with greater baseline severity. This selection bias could potentially lead to an underestimation or misinterpretation of the true therapeutic effect. Although we adjusted for mechanical ventilation as a proxy for severity in our GLMM analysis, the lack of standardized functional metrics (e.g., GBS disability scores) in original case reports limits our ability to fully account for residual confounding from unmeasured severity markers. We hope that future studies will use more refined efficacy measures such as functional recovery scales, long-term assessments, and broader outcome indicators, to better capture the dynamic nature of treatment responses, promoting earlier recovery and preventing further deterioration. Third, the use of case reports and small series may have introduced some publication bias and limited generalizability. Fourth, the binary classification of efficacy (effective *vs*. ineffective) may not fully capture the complexity of treatment outcomes, particularly regarding gradual improvement and individual variability in recovery time. Treatment for GBS often involves progressive recovery, which may not be fully reflected in a simplified binary approach. While this categorization allows for practical comparisons across studies, it may not encompass the nuances of individual patient responses. Despite these limitations, our findings offer an exploratory synthesis to guide future prospective studies with more rigorous designs. We sincerely hope that future prospective studies with standardized designs and follow-up protocols will become available to provide more definitive conclusions.

## 5 Conclusion

In conclusion, this systematic review and exploratory analysis suggest that for patients with CTDs-GBS, intensive combination regimens particularly those incorporating both glucocorticoids and immunosuppressants (GC + IS) demonstrate potential advantages in improving short-term neurological outcomes compared to traditional IVIG monotherapy. While our GLMM analysis observed a statistically significant association for certain regimens, these findings should be interpreted with caution as hypothesis-generating clues rather than definitive clinical guidelines, given the inherent limitations of retrospective case reports, the wide confidence intervals, and the potential for confounding by indication. Future large-scale, prospective cohort studies utilizing standardized functional assessment scales and fixed follow-up protocols are urgently required to further validate the hypotheses proposed in this study. Such efforts will be instrumental in establishing optimized treatment pathways tailored to various CTD subtypes and GBS variants, thereby improving the long-term prognosis of patients with this complex, interdisciplinary disease.

## Data Availability

The original contributions presented in the study are included in the article/**Supplementary Material**. Further inquiries can be directed to the corresponding authors.
