# Supplementary material for: Qualitative evaluation of pharmacological strategy for connective tissue diseases with Guillain-Barré syndrome: a systematic review
Source: Front Immunol. 2026 Mar 18;17:1684941. doi: 10.3389/fimmu.2026.1684941 (PMC13047961; doi:10.3389/fimmu.2026.1684941)
Supplement: Supplementary file 1 [file Table1.docx]

search strategy:

Pubmed: (("Guillain-Barre Syndrome"[Mesh]) OR ((((((((((((Guillain–Barre Syndrome) OR (Guillain–Barre)) OR (Guillain–Barré Syn-drome)) OR (acute autoimmune neuropathy)) OR (acute inflammatory demyelinating polyneuropathy)) OR (acute inflammatory demyelinating polyradiculoneuropathy)) OR (acute inflammatory polyneuropathy)) OR (Miller–Fisher)) OR (AMSAN)) OR (AIDP)) OR (AMAN)) OR (GBS))) AND (("Connective Tissue Diseases"[Mesh]) OR (((Connective Tissue Disease[Title/Abstract]) OR (Disease, Connective Tissue[Title/Abstract])) OR (Diseases, Connective Tissue[Title/Abstract])))

Embase:

| **#7** | #3 AND #6 |
| --- | --- |
| **#6** | #4 OR #5 |
| **#5** | 'guillain–barre syndrome':ab,ti OR 'guillain barre disease':ab,ti OR 'guillain barre polyradiculitis':ab,ti OR 'inflammatory acute polyradiculoneuropathy':ab,ti OR 'miller fisher syndrome':ab,ti OR 'acute autoimmune neuropathy':ab,ti OR 'acute inflammatory demyelinating polyneuropathy':ab,ti OR 'acute inflammatory demyelinating polyradiculoneuropathy':ab,ti OR aman:ab,ti OR amsan:ab,ti OR 'guillain–barré syndrome':ab,ti |
| **#4** | 'guillain barre syndrome'/exp |
| **#3** | #1 OR #2 |
| **#2** | 'connective tissue disorder':ab,ti OR 'connective tissue defect':ab,ti OR 'connective tissue diseases':ab,ti OR 'connective tissue dysplasia':ab,ti OR 'mesenchymal disease':ab,ti OR 'connective tissue disease':ab,ti |
| **#1** | 'connective tissue disease'/exp |

Table S1. Qualitative assessment of the included studies.

|  | Domains for Evaluating the Methodological Quality of Case Reports and Case Series | | | | | | | |  |
| --- | --- | --- | --- | --- | --- | --- | --- | --- | --- |
|  | Selection | Ascertainment | | Causality | | | | Reporting |  |
| References | Question 1 | Question 2 | Question 3 | Question 4 | Question 5 | Question 6 | Question 7 | Question 8 |  |
| jZhang, et al. [1] | Yes | Yes | Yes | Yes | No | Yes | No | Yes | High |
| Yu and Geraldi-Samara [2] | Yes | Yes | Yes | Yes | No | No | No | Yes | High |
| Yamamoto, et al. [3] | Yes | Yes | Yes | Yes | No | No | No | yes | High |
| Wańkowicz, et al. [4] | Yes | Yes | Yes | Yes | No | No | Yes | yes | High |
| Velázquez Benito, et al. [5] | Yes | Yes | Yes | Yes | No | No | No | Yes | High |
| Tanaka, et al. [6] | Yes | Yes | Yes | Yes | No | No | Yes | Yes | High |
| Su, et al. [7] | Yes | Yes | Yes | Yes | No | No | No | Yes | High |
| Santos, et al. [8] | Yes | Yes | Yes | Yes | No | No | Yes | Yes | High |
| Nadri and Althaf [9] | Yes | Yes | Yes | Yes | No | No | Yes | Yes | High |
| Mochizuki, et al. [10] | Yes | Yes | Yes | Yes | No | No | Yes | Yes | High |
| Matsuki, et al. [11] | Yes | Yes | Yes | Yes | No | No | Yes | No | Low |
| Kumar, et al. [12] | Yes | Yes | Yes | Yes | No | No | Yes | Yes | High |
| Gao, et al. [13] | Yes | Yes | Yes | Yes | No | No | Yes | Yes | High |
| Fazio, et al. [14] | Yes | Yes | Yes | Yes | No | No | Yes | Yes | High |
| Dick and Raman [15] | Yes | Yes | Yes | Yes | No | No | No | No | Low |
| Çoban, et al. [16] | Yes | Yes | Yes | Yes | No | No | No | Yes | High |
| Chen and Su [17] | Yes | Yes | Yes | Yes | No | No | Yes | Yes | High |
| Chaudhuri, et al. [18] | Yes | Yes | Yes | Yes | No | No | Yes | No | Low |
| Boudanga, et al. [19] | Yes | Yes | Yes | Yes | No | No | No | yes | High |
| Bingisser, et al. [20] | Yes | Yes | Yes | Yes | No | No | Yes | Yes | High |
| Awad, et al. [21] | Yes | Yes | Yes | Yes | No | No | No | Yes | High |
| Anyfantakis, et al. [22] | Yes | Yes | Yes | Yes | No | No | No | Yes | High |
| Alvarez-Lario, et al. [23] | Yes | Yes | Yes | Yes | No | No | Yes | Yes | High |
| Akgun, et al. [24] | Yes | Yes | Yes | Yes | No | No | No | Yes | High |
| Roquer, et al. [25] | Yes | Yes | Yes | Yes | No | No | No | Yes | High |
| Xie [26] | Yes | Yes | Yes | Yes | No | No | No | yes | High |
| van Laarhoven, et al. [27] | Yes | Yes | Yes | Yes | No | No | No | Yes | High |
| Beshir, et al. [28] | Yes | Yes | Yes | Yes | No | No | No | yes | High |
| Braun, et al. [29] | Yes | Yes | Yes | Yes | No | No | No | yes | High |
| Hsu, et al. [30] | Yes | Yes | Yes | Yes | No | No | Yes | Yes | High |
| Kopanidis, et al. [31] | Yes | Yes | Yes | Yes | No | No | No | Yes | High |
| Lee, et al. [32] | Yes | Yes | Yes | Yes | No | No | Yes | Yes | High |
| Lewis and Gibson [33] | Yes | Yes | Yes | Yes | No | No | Yes | Yes | High |
| López Méndez, et al. [34] | Yes | Yes | Yes | Yes | No | No | No | Yes | High |
| Rajadhyaksha and Mehra [35] | Yes | Yes | Yes | Yes | No | No | No | No | Low |
| Santiago-Casas, et al. [36] | Yes | Yes | Yes | Yes | No | No | No | Yes | High |
| Rajeshwari, et al. [37] | Yes | Yes | Yes | Yes | No | No | No | Yes | High |
| Stahl, et al. [38] | Yes | Yes | Yes | Yes | No | No | Yes | Yes | High |
| Millette, et al. [39] | Yes | Yes | Yes | Yes | No | No | No | No | Low |
| Moreira Filho, et al. [40] | Yes | Yes | Yes | Yes | No | No | No | Yes | High |
| Wang, et al. [41] | Yes | Yes | Yes | Yes | No | No | No | No | Low |
| Ubogu, et al. [42] | yes | yes | yes | yes | no | no | no | yes | High |
| Thakolwiboon, et al. [43] | yes | yes | yes | Yes | No | No | yes | Yes | High |
| Robson, et al. [44] | yes | yes | yes | yes | No | no | no | Yes | High |
| Sangani, et al. [45] | yes | yes | yes | yes | No | No | yes | yes | High |
| Okoh, et al. [46] | yes | yes | yes | yes | No | No | no | yes | High |
| Meher, et al. [47] | yes | yes | yes | yes | no | no | no | yes | High |
| Javadi Parvaneh, et al. [48] | yes | yes | Yes | yes | no | no | yes | Yes | High |
| Coomes, et al. [49] | yes | yes | yes | yes | No | no | no | yes | High |
| Basnet [50] | yes | yes | yes | yes | No | no | yes | yes | High |
| Althagafi, et al. [51] | yes | yes | yes | yes | No | no | no | yes | High |
| Lin, et al. [52] | yes | yes | yes | yes | no | no | Yes | Yes | High |
| Ethemoglu, et al. [53] | yes | yes | Yes | yes | no | no | yes | yes | High |
| Ali, et al. [54] | yes | no | yes | yes | no | no | yes | Yes | High |
| Vaidya, et al. [55] | yes | no | yes | yes | no | no | yes | No | Low |
| Miyagawa, et al. [56] | yes | yes | yes | yes | No | no | no | yes | High |
| Mouti, et al. [57] | yes | yes | Yes | yes | No | No | no | Yes | High |
| Brahmandam, et al. [58] | yes | yes | yes | yes | no | no | no | yes | High |
| Ernst, et al. [59] | yes | yes | yes | yes | no | no | no | yes | High |
| Sainatham, et al. [60] | yes | yes | yes | yes | no | no | yes | yes | High |
| Gacem, et al. [61] | yes | yes | yes | yes | no | no | no | Yes | High |
| Bencivenga, et al. [62] | yes | yes | yes | yes | no | no | yes | Yes | High |
| Alhariri, et al. [63] | Yes | Yes | Yes | Yes | No | No | Yes | Yes | High |
| Mehta, et al. [64] | Yes | Yes | Yes | Yes | No | No | No | Yes | High |
| Salem, et al. [65] | Yes | Yes | Yes | Yes | No | No | Yes | Yes | High |
| Garisa and Thiruchelvam [66] | Yes | Yes | Yes | Yes | No | No | Yes | Yes | High |
| Akgun and Wu [67] | Yes | Yes | Yes | Yes | No | No | No | Yes | High |
| Yew, et al. [68] | Yes | Yes | Yes | Yes | No | No | No | Yes | High |
| Hatami, et al. [69] | Yes | Yes | Yes | Yes | No | No | yes | Yes | High |
| Koryllou, et al. [70] | Yes | Yes | Yes | Yes | No | No | yes | Yes | High |
| Entz, et al. [71] | Yes | Yes | Yes | Yes | No | No | no | yes | High |
| Coomes and Haghbayan [72] | Yes | Yes | Yes | Yes | No | No | yes | yes | High |
| Thakolwiboon and Karukote [73] | Yes | Yes | Yes | Yes | No | No | yes | Yes | High |
| Arain, et al. [74] | Yes | Yes | Yes | Yes | No | No | No | Yes | High |
| Wall and Katz [75] | yes | yes | yes | yes | no | no | no | yes | High |
| Chandrashekhar and De Sousa [76] | yes | yes | yes | Yes | No | No | yes | Yes | High |
| De Araujo, et al. [77] | yes | yes | yes | yes | No | no | no | yes | High |
| Ogawa, et al. [78] | yes | yes | yes | yes | no | No | yes | yes | High |
| Pérez-García, et al. [79] | yes | yes | yes | yes | No | No | no | yes | High |
| Adams, et al. [80] | yes | yes | yes | no | no | no | no | No | Low |
| Tantoush, et al. [81] | yes | yes | Yes | yes | no | no | yes | Yes | High |
| Güzelay, et al. [82] | yes | yes | yes | yes | No | no | no | No | Low |
| Aslam, et al. [83] | yes | yes | yes | yes | No | no | yes | yes | High |
| Landete and Blasco [84] | yes | yes | yes | yes | No | no | no | yes | High |
| Karaca, et al. [85] | yes | yes | yes | yes | no | no | no | yes | High |
| Smyth and Jacobson [86] | yes | yes | Yes | yes | no | no | yes | yes | High |
| Ghiringhelli, et al. [87] | yes | no | yes | yes | no | no | yes | Yes | High |
| Anderson, et al. [88] | yes | no | yes | yes | no | no | yes | No | Low |
| Nazir, et al. [89] | yes | yes | yes | yes | No | No | no | yes | High |
| Chabchoub, et al. [90] | yes | yes | yes | yes | no | no | yes | yes | High |
| Yamaguchi, et al. [91] | yes | yes | Yes | yes | no | no | yes | Yes | High |
| de la Cámara Fernández, et al. [92] | yes | yes | yes | yes | No | no | no | yes | High |
| Das and Sarkar [93] | yes | yes | yes | yes | No | no | no | yes | High |
| Saied, et al. [94] | yes | yes | yes | yes | no | no | yes | yes | High |
| B. Levinson, et al. [95] | yes | yes | Yes | yes | no | no | no | No | Low |
|  |  |  |  |  |  |  |  |  |  |

Selection:

1. Does the patient(s) represent(s) the whole experience of the investigator (center) or is the selection method unclear to the extent that other patients with similar presentation may not have been reported Ascertainment:

2. Was the exposure adequately ascertained？

3. Was the outcome adequately ascertained？

Causality:

4. Were other alternative causes that may explain the observation ruled out？

5. Was there a challenge/rechallenge phenomenon？

6. Was there a dose-response effect?

7. Was follow-up long enough for outcomes to occur?

Reporting:

8. Is the case(s) described with sufficient details to allow other investigators to replicate the research or to allow practitioners make inferences related to their own practice?

These studies’ countries include China (8 cases), Japan (6 cases), Morocco (2 cases), the United Kingdom (4 cases), the United States (28 cases), Algeria (1 case), Arab countries (3 cases), Ireland (1 case), Australia (1 case), Pakistan (2 cases), Brazil (3 cases), Poland (1 case), Germany (2 cases), France (1 case), South Korea (1 case), the Netherlands (2 cases), Canada (2 cases), the Maldives (1 case), Malaysia (1 case), Bangladesh (1 case), Mexico (1 case), Switzerland (2 cases), Tunisia (1 case), Turkey (3 cases), Spain (6 cases), Greece (1 case), Italy (2 cases), and India (8 cases).

Table S2. Characteristics of included patients

| Year | Patient ID | Age | Sex | CTD type | GBS type | Treatment Sequence | Treatment Regimen | Outcome | Mechanical ventilation |
| --- | --- | --- | --- | --- | --- | --- | --- | --- | --- |
| 2017 | 1 | 65 | 1 | SLE | NA | 1 | IVIG | 0 | 1 |
| 2017 | 1 | 65 | 1 | SLE | NA | 2 | GC | 1 | 1 |
| 2020 | 2 | 55 | 1 | SS | MFS | 1 | IVIG | 1 | 0 |
| 2015 | 3 | 49 | 1 | SS | NA | 1 | IVIG | 0 | 0 |
| 2015 | 3 | 49 | 1 | SS | NA | 2 | GC+IVIG | 1 | 0 |
| 2018 | 4 | 41 | 1 | SLE | AMAN | 1 | GC | 0 | 0 |
| 2018 | 4 | 41 | 1 | SLE | AMAN | 2 | PE+IVIG | 1 | 0 |
| 2014 | 5 | 62 | 1 | RA | AMSAN | 1 | PE+GC | 1 | 0 |
| 2016 | 6 | 57 | 1 | SS | AIDP | 1 | IVIG | 0 | 0 |
| 2016 | 6 | 57 | 1 | SS | AIDP | 2 | GC | 1 | 0 |
| 2017 | 7 | 26 | 1 | SLE | NA | 1 | GC+IVIG+IS | 1 | 0 |
| 2009 | 8 | 28 | 1 | SLE | AMSAN | 1 | GC | 0 | 1 |
| 2009 | 8 | 28 | 1 | SLE | AMSAN | 2 | PE+IVIG | 0 | 1 |
| 2015 | 9 | 23 | 1 | SLE | NA | 1 | PE+GC+IVIG | 1 | 0 |
| 2002 | 10 | 67 | 1 | SS | AMAN | 1 | GC | 0 | 0 |
| 2002 | 10 | 67 | 1 | SS | AMAN | 2 | IVIG | 1 | 0 |
| 1999 | 11 | 61 | 1 | SLE | NA | 1 | GC+IS | 1 | 0 |
| 2019 | 12 | 41 | 1 | SS | AIDP | 1 | PE+GC | 1 | 0 |
| 2018 | 13 | 30 | 1 | SLE | AIDP | 1 | GC+IS | 1 | 0 |
| 2015 | 14 | 44 | 1 | SLE | AIDP | 1 | IVIG | 0 | 0 |
| 2015 | 14 | 44 | 1 | SLE | AIDP | 2 | IS | 1 | 0 |
| 1982 | 15 | 53 | 1 | RA | NA | 1 | GC | 1 | 0 |
| 2016 | 16 | 26 | 1 | SS | AMAN | 1 | IVIG | 1 | 0 |
| 2021 | 17 | 63 | 1 | SS | AMSAN | 1 | GC+IVIG | 1 | 0 |
| 1989 | 18 | 40 | 1 | SLE | AIDP | 1 | PE+GC+IS | 1 | 0 |
| 2023 | 19 | 41 | 1 | SLE | AMAN | 1 | GC+IS | 1 | 1 |
| 1994 | 20 | 17 | 1 | SLE | MFS | 1 | IVIG | 0 | 1 |
| 1994 | 20 | 17 | 1 | SLE | MFS | 2 | PE+GC+IS | 1 | 0 |
| 2010 | 21 | 26 | 1 | SS | AMAN | 1 | PE | 0 | 1 |
| 2010 | 21 | 26 | 1 | SS | AMAN | 2 | IVIG | 1 | 1 |
| 2014 | 22 | 77 | 1 | SLE | AMSAN | 1 | GC+IVIG | 0 | 1 |
| 2014 | 22 | 77 | 1 | SLE | AMSAN | 2 | GC | 0 | 1 |
| 2013 | 23 | 50 | 1 | RA | AMAN | 1 | GC+IS | 0 | 1 |
| 2013 | 23 | 50 | 1 | RA | AMAN | 2 | IVIG | 0 | 1 |
| 2022 | 24 | 20 | 1 | SLE | AMAN | 1 | GC+IVIG | 0 | 1 |
| 2022 | 24 | 20 | 1 | SLE | AMAN | 2 | PE+IS | 1 | 1 |
| 2022 | 25 | 37 | 1 | SLE | AIDP | 1 | IVIG | 0 | 1 |
| 2022 | 25 | 37 | 1 | SLE | AIDP | 2 | PE+GC+IS | 1 | 1 |
| 1985 | 26 | 50 | 1 | RA | MFS | 1 | GC | 1 | 0 |
| 2024 | 27 | 40 | 1 | SLE | NA | 1 | PE+GC+IS | 0 | 1 |
| 2001 | 28 | 20 | 1 | SLE | NA | 1 | IVIG | 0 | 1 |
| 2001 | 28 | 20 | 1 | SLE | NA | 2 | GC+IS | 1 | 1 |
| 2022 | 29 | 14 | 1 | SLE | AMAN | 1 | IVIG | 0 | 1 |
| 2022 | 29 | 14 | 1 | SLE | AMAN | 2 | GC | 0 | 1 |
| 2022 | 29 | 14 | Sex | SLE | AMAN | 3 | PE | 1 | 1 |
| 2024 | 30 | 34 | 1 | SLE | AMSAN | 1 | IVIG | 0 | 1 |
| 2024 | 30 | 34 | 1 | SLE | AMSAN | 2 | PE+IVIG | 1 | 1 |
| 2024 | 30 | 34 | 1 | SLE | AMSAN | 3 | PE+GC+IVIG | 1 | 1 |
| 2009 | 31 | 28 | 1 | SLE | AIDP | 1 | PE+GC+IS | 1 | 0 |
| 2021 | 32 | 37 | 1 | SLE | AIDP | 1 | IVIG | 0 | 0 |
| 2021 | 32 | 37 | 1 | SLE | AIDP | 2 | PE+GC | 1 | 0 |
| 2023 | 33 | 50 | 1 | SLE | AMAN | 1 | GC | 0 | 0 |
| 2023 | 33 | 50 | 1 | SLE | AMAN | 2 | GC+IVIG | 1 | 0 |
| 2003 | 34 | 21 | 1 | SLE | AIDP | 1 | IS | 0 | 0 |
| 2003 | 34 | 21 | 1 | SLE | AIDP | 2 | GC+IVIG+IS | 0 | 0 |
| 2003 | 34 | 21 | 1 | SLE | AIDP | 3 | IVIG | 1 | 0 |
| 2010 | 35 | 31 | 1 | RA | AIDP | 1 | GC+IVIG | 1 | 0 |
| 2012 | 36 | 30 | 1 | SLE | AMAN | 1 | GC+IVIG+IS | 1 | 0 |
| 2013 | 37 | 20 | 1 | SLE | AMAN | 1 | PE | 0 | 1 |
| 2013 | 37 | 20 | 1 | SLE | AMAN | 2 | IVIG | 0 | 1 |
| 2013 | 37 | 20 | 1 | SLE | AMAN | 3 | GC+IS | 1 | 1 |
| 2013 | 38 | 34 | 1 | SLE | AMSAN | 1 | GC+IVIG+IS | 1 | 0 |
| 2016 | 39 | 20 | 1 | SLE | AMSAN | 1 | PE+GC | 1 | 0 |
| 2000 | 40 | 56 | 1 | SLE | AIDP | 1 | GC | 0 | 1 |
| 2000 | 40 | 56 | 1 | SLE | AIDP | 2 | IVIG | 0 | 1 |
| 2000 | 40 | 56 | 1 | SLE | AIDP | 3 | GC+IS | 1 | 1 |
| 1986 | 41 | 23 | 1 | SLE | NA | 1 | GC+IVIG | 1 | 0 |
| 1980 | 42 | 22 | 1 | SLE | NA | 1 | GC | 1 | 1 |
| 2024 | 43 | 29 | 1 | SLE | NA | 1 | GC+IVIG+IS | 1 | 0 |
| 2024 | 44 | 44 | 1 | SLE | NA | 2 | GC+IVIG | 1 | 0 |
| 2024 | 45 | 62 | 1 | SLE | NA | 3 | GC+IVIG+IS | 0 | 0 |
| 2024 | 46 | 15 | 1 | SLE | NA | 4 | GC+IVIG+IS | 1 | 0 |
| 2024 | 47 | 21 | 1 | SLE | NA | 5 | GC+IVIG+IS | 1 | 0 |
| 2024 | 48 | 24 | 1 | SLE | NA | 6 | GC+IVIG+IS | 1 | 0 |
| 2001 | 49 | 27 | 1 | SLE | AMSAN | 1 | GC | 0 | 1 |
| 2001 | 49 | 27 | 1 | SLE | AMSAN | 2 | GC+IS | 0 | 1 |
| 2001 | 49 | 27 | 1 | SLE | AMSAN | 3 | IVIG | 1 | 1 |
| 2019 | 50 | 72 | 1 | SLE | AMSAN | 1 | IVIG | 0 | 0 |
| 2019 | 50 | 72 | 1 | SLE | AMSAN | 2 | PE+GC+IS | 1 | 0 |
| 1994 | 51 | 33 | 1 | SLE | AIDP | 1 | GC | 0 | 0 |
| 1994 | 51 | 33 | 1 | SLE | AIDP | 2 | GC+IS | 0 | 0 |
| 2021 | 52 | 80 | 1 | SLE | NA | 1 | GC | 0 | 0 |
| 2021 | 52 | 80 | 1 | SLE | NA | 2 | IVIG | 1 | 0 |
| 2015 | 53 | 41 | 1 | SLE | MFS | 1 | PE | 0 | 0 |
| 2015 | 53 | 41 | 1 | SLE | MFS | 2 | GC | 0 | 0 |
| 2015 | 53 | 41 | 1 | SLE | MFS | 3 | PE+GC+IS | 0 | 0 |
| 2015 | 53 | 41 | 1 | SLE | MFS | 4 | GC+IVIG | 1 | 0 |
| 2024 | 54 | 17 | 1 | SLE | AIDP | 1 | PE+GC+IS | 1 | 1 |
| 2020 | 55 | 16 | 1 | SLE | MFS | 1 | GC+IVIG | 0 | 0 |
| 2020 | 55 | 16 | 0 | SLE | MFS | 2 | PE+GC+IS | 1 | 0 |
| 2018 | 56 | 45 | 1 | SLE | AMSAN | 1 | IVIG | 0 | 1 |
| 2018 | 56 | 45 | 1 | SLE | AMSAN | 2 | PE+GC+IS | 1 | 1 |
| 2023 | 57 | 22 | 1 | SLE | AMSAN | 1 | GC+IVIG+IS | 0 | 1 |
| 2023 | 57 | 22 | 1 | SLE | AMSAN | 2 | PE+IS | 1 | 1 |
| 2023 | 58 | 20 | 1 | SLE | AMAN | 1 | GC | 0 | 1 |
| 2023 | 58 | 20 | 1 | SLE | AMAN | 2 | GC+IVIG+IS | 1 | 1 |
| 2024 | 59 | 57 | 1 | SS | AIDP | 1 | IVIG | 0 | 0 |
| 2024 | 59 | 57 | 1 | SS | AIDP | 2 | PE | 0 | 0 |
| 2024 | 59 | 57 | 1 | SS | AIDP | 3 | GC+IS | 1 | 0 |
| 2016 | 60 | 82 | 1 | SS | AIDP | 1 | PE | 0 | 1 |
| 2016 | 60 | 82 | 1 | SS | AIDP | 2 | IVIG+IS | 1 | 1 |
| 2018 | 61 | 42 | 1 | SS | AMSAN | 1 | IVIG | 0 | 0 |
| 2018 | 61 | 42 | 1 | SS | AMSAN | 2 | GC+IS | 1 | 0 |
| 2017 | 62 | 38 | 1 | SLE | AIDP | 1 | PE+GC | 0 | 0 |
| 2017 | 62 | 38 | 1 | SLE | AIDP | 2 | GC+IVIG+IS | 1 | 0 |
| 1999 | 63 | 23 | 1 | SLE | AIDP | 1 | PE+GC+IS | 1 | 1 |
| 2000 | 64 | 13 | 1 | SS | NA | 1 | PE+GC+IVIG | 1 | 0 |
| 2002 | 65 | 25 | 1 | SLE | NA | 1 | GC+IVIG | 0 | 0 |
| 2024 | 66 | 28 | 1 | SLE | AIDP | 1 | GC+IVIG | 0 | 1 |
| 2024 | 66 | 28 | 1 | SLE | AIDP | 2 | PE | 0 | 1 |
| 2024 | 66 | 28 | 1 | SLE | AIDP | 3 | GC+IS | 1 | 1 |
| 2024 | 67 | 75 | 1 | SLE | AIDP | 1 | PE | 0 | 1 |
| 2024 | 67 | 75 | 1 | SLE | AIDP | 2 | GC+IS | 1 | 1 |
| 2023 | 68 | 23 | 1 | SLE | AMSAN | 1 | GC | 0 | 1 |
| 2023 | 68 | 23 | 1 | SLE | AMSAN | 2 | PE | 1 | 1 |
| 2023 | 69 | 13 | 1 | SLE | NA | 1 | GC+IVIG | 0 | 0 |
| 2023 | 70 | 75 | 1 | RA | NA | 1 | IVIG | 0 | 0 |
| 2023 | 70 | 75 | 1 | RA | NA | 2 | GC | 1 | 0 |
| 2023 | 71 | 44 | 1 | MCTD | AMSAN | 1 | IVIG | 0 | 1 |
| 2023 | 72 | 21 | 1 | SLE | NA | 1 | PE+GC | 0 | 1 |
| 2023 | 72 | 21 | 1 | SLE | NA | 2 | IVIG | 0 | 1 |
| 2023 | 72 | 21 | 1 | SLE | NA | 3 | GC | 0 | 1 |
| 2023 | 72 | 21 | 1 | SLE | NA | 4 | GC+IS | 1 | 1 |
| 2022 | 73 | 67 | 1 | MCTD | AMSAN | 1 | GC+IVIG | 1 | 1 |
| 2022 | 74 | 67 | 1 | SLE | AMSAN | 1 | PE+GC+IVIG | 0 | 1 |
| 2022 | 74 | 67 | 1 | SLE | AMSAN | 2 | GC+IS | 1 | 1 |
| 2021 | 75 | 21 | 1 | SLE | AMAN | 1 | GC+IVIG | 0 | 1 |
| 2021 | 75 | 21 | 1 | SLE | AMAN | 2 | PE+IS | 1 | 1 |
| 2020 | 76 | 64 | 1 | SLE | AMSAN | 1 | GC+IVIG | 0 | 0 |
| 2020 | 77 | 22 | 1 | SLE | AIDP | 1 | IVIG | 0 | 0 |
| 2020 | 77 | 22 | 1 | SLE | AIDP | 2 | GC+IS | 1 | 0 |
| 2020 | 78 | 11 | 1 | SLE | AMAN | 1 | PE+GC+IVIG+IS | 1 | 1 |
| 2019 | 79 | 29 | 1 | SLE | AMSAN | 1 | PE+IVIG | 0 | 1 |
| 2019 | 80 | 45 | 1 | SLE | AMSAN | 1 | IVIG | 0 | 1 |
| 2019 | 80 | 45 | 0 | SLE | AMSAN | 2 | PE+GC+IS | 1 | 1 |
| 2019 | 81 | 72 | 1 | SLE | AMSAN | 1 | IVIG | 0 | 0 |
| 2019 | 81 | 72 | 1 | SLE | AMSAN | 2 | PE+GC+IS | 1 | 0 |
| 2018 | 82 | 24 | 1 | PM | AMSAN | 1 | PE+GC+IS | 0 | 0 |
| 2018 | 83 | 32 | 1 | SLE | NA | 1 | PE+IVIG | 0 | 1 |
| 2018 | 83 | 32 | 1 | SLE | NA | 2 | GC+IS | 1 | 1 |
| 2018 | 84 | 18 | 1 | SLE | NA | 1 | PE+GC | 1 | 1 |
| 2018 | 85 | 19 | 1 | SLE | AMSAN | 1 | GC | 0 | 0 |
| 2018 | 85 | 19 | 1 | SLE | AMSAN | 2 | IVIG | 0 | 0 |
| 2018 | 85 | 19 | 1 | SLE | AMSAN | 3 | GC+IVIG+IS | 1 | 0 |
| 2017 | 86 | 31 | 1 | SLE | MFS | 1 | IVIG | 0 | 0 |
| 2017 | 86 | 31 | 1 | SLE | MFS | 2 | GC+IS | 1 | 0 |
| 2016 | 87 | 18 | 1 | SLE | NA | 1 | IVIG | 0 | 0 |
| 2016 | 87 | 18 | 1 | SLE | NA | 2 | GC+IS | 1 | 0 |
| 2016 | 88 | 67 | 1 | SS | AIDP | 1 | IVIG | 0 | 0 |
| 2016 | 88 | 67 | 1 | SS | AIDP | 2 | PE+GC+IS | 0 | 0 |
| 2014 | 89 | 59 | 1 | SLE | AIDP | 1 | GC+IVIG | 0 | 0 |
| 2014 | 89 | 59 | 1 | SLE | AIDP | 2 | GC+IS | 1 | 0 |
| 2013 | 90 | 32 | 1 | SLE | NA | 1 | IVIG | 0 | 1 |
| 2013 | 90 | 32 | 1 | SLE | NA | 1 | PE+GC | 1 | 1 |
| 2012 | 91 | 61 | 1 | SLE | AMSAN | 1 | IVIG | 0 | 0 |
| 2012 | 91 | 61 | 1 | SLE | AMSAN | 2 | GC+IS | 1 | 0 |
| 1998 | 92 | 28 | 1 | SS | AIDP | 1 | GC | 1 | 0 |
| 2018 | 93 | 40 | 1 | SS | NA | 1 | GC+IVIG | 1 | 0 |
| 2019 | 94 | 41 | 1 | SS | NA | 1 | PE+GC | 1 | 0 |
| 2014 | 95 | 40 | 1 | SS | NA | 1 | PE+IS | 1 | 0 |
| 2014 | 96 | 39 | 1 | SS | NA | 1 | PE+GC+IS | 1 | 0 |
| 2014 | 97 | 64 | 1 | SS | NA | 1 | GC+IVIG+IS | 1 | 0 |
| 2020 | 98 | 19 | 1 | SLE | AIDP | 1 | PE+IS | 1 | 1 |
| 2020 | 99 | 37 | 1 | SLE | AIDP | 1 | PE+IS | 1 | 0 |
| 2011 | 100 | 33 | 1 | SLE | NA | 1 | GC | 0 | 0 |
| 2011 | 100 | 33 | 1 | SLE | NA | 2 | PE | 1 | 0 |
| 2010 | 101 | 52 | 1 | PM | AIDP | 1 | GC+IVIG | 0 | 0 |
| 2024 | 102 | 30 | 1 | RA | AMSAN | 1 | PE+GC+IS | 1 | 0 |
| 2022 | 103 | 19 | 1 | PM | NA | 1 | PE | 0 | 1 |
| 2022 | 103 | 19 | 1 | PM | NA | 2 | GC+IS | 1 | 1 |
| 2000 | 104 | 51 | 1 | PM | NA | 1 | GC | 0 | 1 |
| 2000 | 104 | 51 | 1 | PM | NA | 2 | PE+GC | 1 | 1 |
| 2018 | 105 | 45 | 1 | SLE | AIDP | 1 | GC+IVIG | 0 | 1 |
| 2018 | 105 | 45 | 1 | SLE | AIDP | 2 | PE | 0 | 1 |

GBS: Guillain–Barre Syndrome. AIDP: Acute Inflammatory Demyelinating Polyneuropathy, AMAN: Acute Motor Axonal Neuropathy, AMSAN: Acute Motor and Sensory Axonal Neuropathy, MFS: Miller Fisher Syndrome, IVIG: Intravenous immunoglobulin, IS: immunosuppressant, PE: Plasma exchange, GC: glucocorticosteroids. CTD: Connective tissue disease. SLE: systemic lupus erythematosus, SS: Sjögren syndrome, RA: rheumatoid arthritis, PM: polymyositis, MCTD: mixed connective tissue disease.

References：

[1] N. Zhang, J. Cao, M. Zhao and L. Sun, The introspection on the diagnosis and treatment process of a case of Guillain-Barré syndrome (GBS) attributed to systemic lupus erythematosus (SLE): A case report, Medicine (Baltimore) 96 (2017) e9037.

[2] E.H. Yu and D. Geraldi-Samara, Pharyngeal-Cervico-Brachial/Miller Fisher Overlap Syndrome With Infliximab Exposure, J Clin Neuromuscul Dis 21 (2020) 157-158.

[3] A. Yamamoto, K. Imai, M. Hamanaka, T. Yamada, H. Yamazaki, K. Tsuto, Y. Tsuji, N. Yamashita and M. Kadoya, [A case of motor dominant neuropathy and focal segmental glomerulosclerosis associated with Sjögren's syndrome], Rinsho Shinkeigaku 55 (2015) 732-6.

[4] P. Wańkowicz, P. Nowacki, M. Brzosko and D. Bobrowska-Snarska, An overlapping case of Bickerstaff brainstem encephalitis and acute motor axonal neuropathy variant of Guillain-Barré syndrome associated with systemic lupus erythematosus, Pol Arch Intern Med 129 (2019) 50-51.

[5] A. Velázquez Benito, E. Bellosta Diago, S. Santos Lasaosa and L.F. Pascual Millán, [Acute sensory-motor axonal neuropathy (Guillain-Barre syndrome) following vertebroplasty], Med Clin (Barc) 143 (2014) 282-3.

[6] K. Tanaka, H. Nakayasu, Y. Suto, S. Takahashi, Y. Konishi, H. Nishimura, R. Ueno, S. Kusunoki and K. Nakashima, Acute Motor-dominant Polyneuropathy as Guillain-Barré Syndrome and Multiple Mononeuropathies in a Patient with Sjögren's Syndrome, Intern Med 55 (2016) 2717-22.

[7] X. Su, X. Qiao, J. Li, L. Gao, C. Wang and L. Wang, Papulonodular mucinosis, Guillain-Barré syndrome and nephrotic syndrome in a patient with systemic lupus erythematosus: a case report, BMC Nephrol 18 (2017) 43.

[8] D.P. Santos, M. Spitz, P. Oliveira, T. Barcia, R.S. Silva, L.L. Azevedo, C. Meilman, L.F. Vasconcellos, C.S. Lacativa and J.N. El-kadum, Overlap of Bickerstaff encephalitis and Guillain-Barré syndrome in a patient with systemic lupus, Arq Neuropsiquiatr 67 (2009) 904-5.

[9] Q. Nadri and M.M. Althaf, Guillian-Barre syndrome as the initial presentation of systemic lupus erythematosus--case report and review of literature, Ann Saudi Med 35 (2015) 263-5.

[10] H. Mochizuki, K. Kamakura, T. Masaki, A. Hirata, R. Nakamura and K. Motoyoshi, Motor dominant neuropathy in Sjögren's syndrome: report of two cases, Intern Med 41 (2002) 142-6.

[11] Y. Matsuki, T. Hidaka, M. Matsumoto, K. Fukushima and K. Suzuki, Systemic lupus erythematosus demonstrating serum anti-GM1 antibody, with sudden onset of drop foot as the initial presentation, Intern Med 38 (1999) 729-32.

[12] N. Kumar, D. Surendran, B.H. Srinivas and C. Bammigatti, Primary Sjogren's syndrome: a great masquerader, BMJ Case Rep 12 (2019).

[13] Z. Gao, X. Li, T. Peng, Z. Hu, J. Liu, J. Zhen and Y. Gao, Systemic lupus erythematosus with Guillian-Barre syndrome: A case report and literature review, Medicine (Baltimore) 97 (2018) e11160.

[14] R.M. Fazio, I. Chen and N. Somal, Guillain-Barré syndrome as first presentation of systemic lupus erythematosus: a rare manifestation complicated by IVIg-induced splenic infarct, BMJ Case Rep 2015 (2015).

[15] D.J. Dick and D. Raman, The Guillain-Barre syndrome following gold therapy, Scand J Rheumatol 11 (1982) 119-20.

[16] A. Çoban, S. Özyurt, K. Meriç, H. Mısırlı, E. Tüzün and R. Türkoğlu, Limbic Encephalitis Associated with Sjögren's Syndrome: Report of Three Cases, Intern Med 55 (2016) 2285-9.

[17] Y.M. Chen and K.Y. Su, Acute motor and sensory axonal neuropathy in association with primary Sjögren's syndrome: a case report, BMC Neurol 21 (2021) 161.

[18] K.R. Chaudhuri, I.K. Taylor, R.M. Niven and R.J. Abbott, A case of systemic lupus erythematosus presenting as Guillain-Barré syndrome, Br J Rheumatol 28 (1989) 440-2.

[19] A. Boudanga, M. Chraa, O.C. Rhazouani and N. Kissani, Severe acute polyradiculoneuritis revealing systemic lupus erythematosus: a case report, Pan Afr Med J 45 (2023) 8.

[20] R. Bingisser, R. Speich, A. Fontana, J. Gmür, B. Vogel and T. Landis, Lupus erythematosus and Miller-Fisher syndrome, Arch Neurol 51 (1994) 828-30.

[21] A. Awad, S. Mathew and B. Katirji, Acute motor axonal neuropathy in association with Sjögren syndrome, Muscle Nerve 42 (2010) 828-30.

[22] D. Anyfantakis, E.K. Symvoulakis, E. Barbounakis, M. Kastanakis, E. Athanasakis, E. Blevrakis and S. Kastanakis, A fatal case of seronegative, late-onset systemic lupus erythematosus presenting with motor sensory axonal polyneuropathy, Mod Rheumatol 24 (2014) 858-61.

[23] B. Alvarez-Lario, R. Prieto-Tejedo, M. Colazo-Burlato and J. Macarrón-Vicente, Severe Guillain-Barré syndrome in a patient receiving anti-TNF therapy. Consequence or coincidence. A case-based review, Clin Rheumatol 32 (2013) 1407-12.

[24] Y. Akgun, J. Langlie, M.A. Huberman and Y. Wu, Therapeutic plasma exchange in a patient with acute motor axonal neuropathy subtype of Guillain-Barre syndrome and systemic lupus erythematosus, J Clin Apher 37 (2022) 405-410.

[25] J. Roquer, J. Herraiz, J. Maymo, A. Olivé and J. Carbonell, Miller-Fisher syndrome (Guillain-Barré syndrome with ophthalmoplegia) during treatment with gold salts in a patient with rheumatoid arthritis, Arthritis Rheum 28 (1985) 838-9.

[26] S. Xie, Systemic lupus erythematosus complicated with Guillain-Barre syndrome: A case report, Asian J Surg 47 (2024) 3666-3667.

[27] H.W. van Laarhoven, F.A. Rooyer, B.G. van Engelen, R. van Dalen and J.H. Berden, Guillain-Barré syndrome as presenting feature in a patient with lupus nephritis, with complete resolution after cyclophosphamide treatment, Nephrol Dial Transplant 16 (2001) 840-2.

[28] E. Beshir, E. Belt, N. Chencheri, A. Saqib, M. Pallavidino, U. Terheggen, A. Abdalla, L. Herlitz, E. Sharif and M. Bitzan, Case Report: Guillain-Barré Syndrome as Primary Presentation of Systemic Lupus Erythematosus (SLE-GBS) in a Teenage Girl, Front Pediatr 10 (2022) 838927.

[29] S. Braun, L. Bastian, C. Hayes, S.C. Owen, C. Craig and A. Nelson, Systemic Lupus Erythematosus Initially Presenting as Acute Motor and Sensory Axonal Neuropathy Variant of Guillain-Barre Syndrome in a Healthy Active Duty Female, Mil Med 189 (2024) e915-e918.

[30] T.Y. Hsu, S.H. Wang, C.F. Kuo, T.F. Chiu and Y.C. Chang, Acute inflammatory demyelinating polyneuropathy as the initial presentation of lupus, Am J Emerg Med 27 (2009) 900.e3-5.

[31] P.C. Kopanidis, A. Kane, M.A. Nguyen and R. Markus, Systemic lupus erythematosus presenting as Guillain-Barré syndrome, Pract Neurol 21 (2021) 237-240.

[32] M. Lee, C.H. Lee, J.Y. Ko and A. Kim, Concomitant Occurrence of Acute Motor Axonal Neuropathy in Systemic Lupus Erythematosus, Am J Phys Med Rehabil 102 (2023) e46-e49.

[33] M. Lewis and T. Gibson, Systemic lupus erythematous with recurrent Guillain-Barré-like syndrome treated with intravenous immunoglobulins, Lupus 12 (2003) 857-9.

[34] P. López Méndez, I. Martín Santana, M. del Pino Reyes Yánez, A. Ruano Hernández, J. Hernández Beriain and M. Hervás García, [Meningeal and Guillain-Barrè syndrome in a patient with rheumatoid arthritis receiving adalimumab therapy], Reumatol Clin 7 (2011) 401-3.

[35] A. Rajadhyaksha and S. Mehra, Pharyngeal-cervical-brachial variant of Guillain-Barre syndrome with predominant bulbar palsy as the initial presentation of systemic lupus erythematosus and lupus nephritis: a case report, Int J Rheum Dis 15 (2012) e162-4.

[36] Y. Santiago-Casas, R.A. Peredo and L.M. Vilá, Efficacy of low-dose intravenous cyclophosphamide in systemic lupus erythematosus presenting with Guillain-Barre syndrome-like acute axonal neuropathies: report of two cases, Lupus 22 (2013) 324-7.

[37] G.K. Rajeshwari, C.H. Rao, N.V. Sundarachary, S.K. Moula Ali, J.S. Kalyani and A. Sridhar, Guillain-Barré syndrome in a patient with neuropsychiatric systemic lupus erythematosus, Natl Med J India 29 (2016) 14-7.

[38] H.D. Stahl, P. Kalischewski, C. Orda, P. Baum, F. Grahmann and F. Emmrich, Filtration of cerebrospinal fluid for acute demyelinating neuropathy in systemic lupus erythematosus, Clin Rheumatol 19 (2000) 61-3.

[39] T.J. Millette, S.H. Subramony, A.S. Wee and V. Harisdangkul, Systemic lupus erythematosus presenting with recurrent acute demyelinating polyneuropathy, Eur Neurol 25 (1986) 397-402.

[40] P.F. Moreira Filho, O.J. Nascimento, D. Cinnicinatus, F.J. Porto, M.R. Freitas and P.C. Santos, [Guillain-Barré syndrome as a manifestation of systemic lupus erythematosus. Report of a case], Arq Neuropsiquiatr 38 (1980) 165-70.

[41] J. Wang, Z. Zhang, J. Qian, S. Zhang, L. Qiao, M. Li, Y. Zhao and X. Zeng, Clinical features of Guillain-Barré syndrome and chronic inflammatory demyelinating polyradiculoneuritis associated with SLE, Lupus Sci Med 11 (2024).

[42] E.E. Ubogu, O.O. Zaidat and J.I. Suarez, Acute motor-sensory axonal neuropathy associated with active systemic lupus erythematosus and anticardiolipin antibodies, J Clin Rheumatol 7 (2001) 326-31.

[43] S. Thakolwiboon, A. Karukote and G. Sohn, Acute motor-sensory axonal neuropathy associated with systemic lupus erythematosus, Proc (Bayl Univ Med Cent) 32 (2019) 610-613.

[44] M.G. Robson, M.J. Walport and K.A. Davies, Systemic lupus erythematosus and acute demyelinating polyneuropathy, Br J Rheumatol 33 (1994) 1074-7.

[45] V. Sangani, M. Pokal, M. Balla, G.P. Merugu, S. Adapa, S. Naramala and V.M. Konala, Pembrolizumab related Guillain barre syndrome, a rare presentation in a patient with a history of lupus and bladder cancer, J Community Hosp Intern Med Perspect 11 (2021) 388-392.

[46] H.C. Okoh, S.S. Lubana, S. Langevin, S. Sanelli-Russo and A. Abrudescu, A Case of Systemic Lupus Erythematosus Presenting as Guillain-Barré Syndrome, Case Rep Rheumatol 2015 (2015) 528026.

[47] J. Meher, J. Singh, S.K. Pati, V.R. Pandit, S.K. Patel, M. Kapur and M.S. Nithin, Deciphering the Neuropsychiatric Lupus Enigma: Navigating the Intersection of Acute Inflammatory Demyelinating Polyneuropathy (AIDP), Infection, and Flare, Cureus 16 (2024) e53156.

[48] V. Javadi Parvaneh, L. Ghasemi, K. Rahmani, R. Shiari, M. Mesdaghi, Z. Chavoshzadeh and S.H. Tonekaboni, Recurrent angioedema, Guillain-Barré, and myelitis in a girl with systemic lupus erythematosus and CD59 deficiency syndrome, Auto Immun Highlights 11 (2020) 9.

[49] E.A. Coomes, H. Haghbayan, J. Spring and S. Mehta, Fulminant Guillain-Barré syndrome in a patient with systemic lupus erythematosus, BMJ Case Rep 12 (2019).

[50] A. Basnet, Systemic lupus erythematosus presenting with moyamoya and Guillain-Barré syndrome, Rheumatol Adv Pract 7 (2023) rkad077.

[51] Z.A. Althagafi, S.S. Al-Bishi, R. Ansari, H.A. Alsolami and L.G. Abdelkader, A Rare Case of Systemic Lupus Erythematosus With Diffuse Alveolar Hemorrhage and Guillain-Barre Syndrome, Cureus 15 (2023) e33984.

[52] Y.K. Lin, F.C. Yang, F.C. Liu, J.T. Lee and Y.F. Sung, Co-Cccurrence of Guillain-Barre Syndrome and Primary Sjögren Syndrome in an Elderly Woman, Acta Neurol Taiwan 25 (2016) 83-87.

[53] O. Ethemoglu, Ö. Kocatürk and E. Zeynep, Acute motor and sensory axonal neuropathy associated with Sjögren's syndrome, Ideggyogy Sz 71 (2018) 352-356.

[54] N. Ali, R. Rampure, F. Malik, S.I. Jafri and D. Amberker, Guillain-Barré syndrome occurring synchronously with systemic lupus erythematosus as initial manifestation treated successfully with low-dose cyclophosphamide, J Community Hosp Intern Med Perspect 6 (2016) 30689.

[55] S. Vaidya, H.E. Jasin and J. Logan, Systemic lupus erythematosus and guillain-barre syndrome, J Clin Rheumatol 5 (1999) 349-53.

[56] S. Miyagawa, M. Nakajima, K. Nishio, J. Sogami, A. Tsubakimoto, A. Yoshioka and T. Shirai, Guillain-Barré syndrome in a child with systemic lupus erythematosus and anti-Ro/SSA and anti-La/SSB autoantibodies, Br J Dermatol 143 (2000) 1050-4.

[57] O. Mouti, H. Harmouch, M. El Alaoui Faris, N. Birouk, S. Kabbaj, S. Aidi, M. Jiddane, W. Maazouzi and T. Chkili, [Acute meningomyelitis and polyradiculoneuritis disclosing systemic lupus erythematosus], Rev Neurol (Paris) 158 (2002) 81-3.

[58] S. Brahmandam, B.S. Brahmandam, S. Brahmandam and S. Brahmandam, GBS: A RARE INITIAL PRESENTATION OF SLE, in: Editor (Ed.)^(Eds.), Book GBS: A RARE INITIAL PRESENTATION OF SLE, 2024, pp. A3018-A3019.

[59] E.B. Ernst, H. Khalil, M. Numeir and S.R. Akbar, Guillain-Barre Syndrome as the First Presentation of Systemic Lupus Erythematosus, American Journal of Respiratory and Critical Care Medicine 209 (2024).

[60] C. Sainatham, H. Mergey Devender, C. Jinka, S. Goel and A. Dilli Babu, GBS IN THE SETTING OF SLE AND DENGUE: A CASE REPORT, Chest 164 (2023) A2640-A2641.

[61] O. Gacem, S. Chabani, Z. Zeroual, Z. Arrada, M. Achir and M.S. Ladj, GUILLAIN-BARRE SYNDROME REVEALING SYSTEMIC LUPUS ERYTHEMATOSUS: A CASE REPORT, Rheumatology (United Kingdom) 62 (2023) iii22-iii23.

[62] R.P. Bencivenga, G. Palumbo, D. Zoppi, E. Cassano, S. Tozza, R. Iodice, R. Dubbioso, L. Ruggiero, M. Nolano and F. Manganelli, TO BBE OR NOT TO BBE: THAT IS THE QUESTION, Journal of the Peripheral Nervous System 28 (2023) S8-S9.

[63] S. Alhariri, M. Hassan, S. Prakash, B. Nguyen and S. Guvvala, Acute motor and sensory axonal neuropathy in a patient with undiagnosed mixed connective tissue disease, American Journal of the Medical Sciences 365 (2023) S388-S389.

[64] P. Mehta, R.L. Luciano, A.M. Aklilu and G.L. Malvar, Guillain-Barre Syndrome Presenting Alongside Lupus Nephritis: Case and Management, Journal of the American Society of Nephrology 34 (2023) 277.

[65] A.M.R. Salem, A.N.A.S. Rihawi, A.M.E.R. Alshekh Mousa, S. Patel, A. A. Salem, M. Tonelli, N. Bates and M. Soliman, AN UNUSUAL CAUSE OF HYPERCARBIC RESPIRATORY FAILURE IN SYSTEMIC SCLEROSIS, Chest 162 (2022) A903-A904.

[66] M. Garisa and N. Thiruchelvam, 'It's Never Lupus': A Case of Rapidly Progressing Polyneuropathy Secondary to Systemic Lupus Erythematosus, American Journal of Respiratory and Critical Care Medicine 205 (2022).

[67] Y. Akgun and Y. Wu, Plasma exchange for acute motor axonal neuropathy variant guillain-barre syndrome in a pregnant woman with active systemic lupus erythematosus, Journal of Clinical Apheresis 36 (2021) 269-270.

[68] J. Yew, C.H. Cheng and M.A.M. Azman, Guillain-Barre Syndrome (GBS) as the initial manifestation of systemic lupus erythematosus (SLE), International Journal of Rheumatic Diseases 23 (2020) 319-320.

[69] M. Hatami, S. Chandra and T. Nguyen, Simultaneous central and peripheral demyelination in a patient with systemic lupus erythematosus, Neurology 94 (2020).

[70] A. Koryllou, M. Mejbri, S. Garcia-Tarodo, P. Parvex, E. Gonzalez Nguyen Tang, C.M. Korff and M. Hofer, Bickerstaff encephalitis with overlapping Guillain-Barré syndrome as a first manifestation of juvenile systemic lupus erythematosus: A case report, Pediatric Rheumatology 18 (2020).

[71] A. Entz, S. Gorgis and J. Uduman, An atypical presentation ofatypical pneumonia: Legionella causing guillain-barre syndrome, Journal of General Internal Medicine 34 (2019) S471.

[72] E. Coomes and H. Haghbayan, LUPUS AND FULMINANT GUILLAIN-BARRÉ SYNDROME, Chest 155 (2019) 101A.

[73] S. Thakolwiboon and A. Karukote, Acute motor-sensory axonal neuropathy associated with systemic lupus erythematosus: A challenge in treatment, Neurology 92 (2019).

[74] S.R. Arain, A. Dahani, S. Sheikh and F. Khan, An unusual case of idiopathic inflammatory polymyositis associated with Guillian Barre syndrome, a management challenge, International Journal of Rheumatic Diseases 21 (2018) 65.

[75] E. Wall and S. Katz, Guillain Barre Syndrome as the first presentation of systemic lupus erythematosus, Journal of Rheumatology 45 (2018) 1011.

[76] S. Chandrashekhar and E. De Sousa, Guillain-barre syndrome as a rare initial presentation of systemic lupus erythematosus, Neurology 90 (2018).

[77] A.A. De Araujo, D.R. De Moura Rodrigues De Aguiar, J.Y.L. De Oliveira, P.G.F. Da Silva, E.S.T.S. De Oliveira, L. De Abreu E Lima Pamplona, L.F.D.P. Miranda and C.B. De Figueiredo, Axonal motor and sensory acute neuropathy in systemic lupus erythematosus: Rare variant of Guillain-Barre syndrome: Case report, Advances in Rheumatology 58 (2018).

[78] E. Ogawa, T. Hoshiyama and J. Okada, Systemic lupus erythematosus presenting as oculomotor disturbance, Lupus Science and Medicine 4 (2017) A106.

[79] J.A. Pérez-García, M. Sáenz-Castro, L.M. González-Galván, P. Arredondo-Ruiz, S. Gutiérrez-Casillas, G. Casas-Aparicio, E. Ramiro-Guerrero, Z. Medina-López, O. Martínez-Mijangos, D. Alonso-Martínez and C.A. Peña-Pérez, Guillain-Barré syndrome as initial manifestation of systemic lupus erythematosus, Medicina Interna de Mexico 32 (2016) 682-687.

[80] D.J. Adams, A. Paraskos, C.J. Rizik, D.J. Boven and V. Narula, Progressive quadriparesis, bulbar dysfunction, and paresthesias in a female with history of sjogren's syndrome: A case report, PM and R 8 (2016) S191.

[81] H.H. Tantoush, A. Abuzaid and H. Al Ashry, When should we consider systemic lupus erythrematosus in patient presents with guillain-barre syndrome, Journal of General Internal Medicine 29 (2014) S469.

[82] N. Güzelay, Z. Uysal Kocabaş, F. Kizilay and S.S. Özkaynak, Guillain-Barre syndrome in systemic lupus erythematosus: Case report, Journal of the Neurological Sciences 333 (2013) e370.

[83] F. Aslam, E.B. Russell and F. Bannout, Cranial nerve palsies: Sarcoidosis to systemic lupus erythematosus, Scandinavian Journal of Rheumatology 41 (2012) 19-20.

[84] L. Landete and R. Blasco, [Sjögren syndrome and subacute demyelinating polyradiculopathy: an unusual association], Rev Neurol 27 (1998) 995-7.

[85] S. Karaca, E.D. Ersözlü Bozkirli, B. Karakurum Göksel, M. Tan and A.E. Yücel, If Neurologists Establish The Diagnosis of Primary Sjogren's Syndrome?, Noro Psikiyatr Ars 51 (2014) 148-156.

[86] D. Smyth and R. Jacobson, Guillain-barre syndrome associated with systemic lupus flare: Two cases and review of the literature, Muscle and Nerve 62 (2020) S35.

[87] P. Ghiringhelli, R. Cattaneo, A. Diana, S. Puricelli and M. Galli, Systemic lupus erythematosus (SLE) associated with antiphospholipid syndrome (APS) and guillain-barré syndrome, European Journal of Internal Medicine 22 (2011) S35.

[88] N. Anderson, K.C. Ho, R. Komorowski, R. Nanchal and J. Durrant, Acute autonomic, sensory, and motor neuropathy with associated autoimmune myositis, Journal of Neuropathology and Experimental Neurology 69 (2010) 556.

[89] Z. Nazir, A. Habib, T. Ali, K. Shafiq Khan, S. Abbas Jaffri and M.A. Haque, Co-occurrence of Guillain–Barre syndrome and rheumatoid arthritis in a young female: A case report from a low middle-income country, Clinical Case Reports 12 (2024).

[90] I. Chabchoub, M. Snoussi, R. Ammar, R. Ben Salah, C. Dammak, F. Frikha, M. Bouaziz, T. Boudawara and Z. Bahloul, About a rare association: Guillain–Barré Syndrome and polymyositis, Clinical Case Reports 10 (2022).

[91] Y. Yamaguchi, Y. Sakurai, T. Mannen and J. Shimizu, Rapidly progressive polymyositis with elevated antiacetylcholine receptor antibody activity, Internal medicine (Tokyo, Japan) 39 (2000) 1108-1110.

[92] I. de la Cámara Fernández, E. Rabadán Rubio and A. de Juanes Montmeterme, A patient with systemic lupus erythematosus with acute inflammatory demyelinating polyradiculoneuropathy with progression to encephalopathy and status epilepticus: A case report, Revista Colombiana de Reumatologia 26 (2019) 201-203.

[93] A. Das and N. Sarkar, Systemic lupus erythematosus presenting as Guillain–Barre syndrome, Annals of Medical Science & Research 1 (2022) 38-40.

[94] Z. Saied, R. Zouari, A. Rachdi, F. Nabli, D. Ben Mohamed and S. Ben Sassi, Cerebral large vessels vasculitis following Guillain-Barré syndrome as first clinical manifestations of primary Sjogren's syndrome: A case based - Review, Heliyon 10 (2024) e30004.

[95] J. B. Levinson, M. Rodriguez Alvarez, K. Koci, A. Feoktistov and I. M. McFarlane, Epstein - Barr virus Infection in a Patient with Neuromyelitis Optica Spectrum Disorder and Sjögren’s Syndrome: A Case Report and Review of Literature, Clinical Case Reports and Reviews 4 (2018).
